# Testing the use of practice facilitation in a cluster randomized stepped-wedge design trial to improve adherence to cardiovascular disease prevention guidelines: HealthyHearts NYC

**DOI:** 10.1186/s13012-016-0450-2

**Published:** 2016-07-04

**Authors:** Donna R. Shelley, Gbenga Ogedegbe, Sheila Anane, Winfred Y. Wu, Keith Goldfeld, Heather T. Gold, Sue Kaplan, Carolyn Berry

**Affiliations:** 1 Department of Population Health, New York University School of Medicine, 227 East 30th Street, 7th floor, New York, NY 10016 USA; 2 Primary Care Information Project, New York City Department of Health and Mental Hygiene, 42-09 28th Street, Long Island City, NY 11101 USA

**Keywords:** Practice facilitation, Primary care, Cardiovascular disease

## Abstract

**Background:**

HealthyHearts NYC (HHNYC) will evaluate the effectiveness of practice facilitation as a quality improvement strategy for implementing the Million Hearts’ ABCS treatment guidelines for reducing cardiovascular disease (CVD) among high-risk patients who receive care in primary care practices in New York City. ABCS refers to (A) aspirin in high-risk individuals; (B) blood pressure control; (C) cholesterol management; and (S) smoking cessation. The long-term goal is to create a robust infrastructure for implementing and disseminating evidence-based practice guidelines (EBPG) in primary care practices.

**Methods/design:**

We are using a stepped-wedge cluster randomized controlled trial design to evaluate the implementation process and the impact of practice facilitation (PF) versus usual care on ABCS outcomes in 250 small primary care practices. Randomization is at the practice site level, all of which begin as part of the control condition. The intervention consists of one year of PF that includes a combination of one-on-one onsite visits and shared learning across practice sites. PFs will focus on helping sites implement evidence-based components of patient-centered medical home (PCMH) and the chronic care model (CCM), which include decision support, provider feedback, self-management tools and resources, and linkages to community-based services.

**Discussion:**

We hypothesize that practice facilitation will result in superior clinical outcomes compared to usual care; that the effects of practice facilitation will be mediated by greater adoption of system changes in accord with PCMH and CCM; and that there will be increased adaptive reserve and change capacity.

**Trial registration:**

NCT02646488

## Background

In 2011, the US Department of Health and Human Services launched the Million Hearts Campaign with an explicit goal of preventing one million heart attacks and strokes by 2017 [1]. A core component of the campaign is to improve implementation and systematic delivery of ABCS treatment guidelines: (A) aspirin in high-risk individuals; (B) blood pressure control; (C) cholesterol management through guideline-recommended use of lipid lowering medications (i.e., statins); and (S) smoking cessation. In order to accelerate adoption of Million Hearts guidelines in primary care practices as well as build capacity for their ongoing implementation and dissemination, the Agency for Healthcare Research and Quality (AHRQ) launched EvidenceNOW—a national initiative that aims to transform health care delivery in small primary care practices by building critical infrastructure to integrate the most up-to-date research into practice to improve the heart health of their patients [2].

There is a strong rationale for targeting small practices (i.e., fewer than 10 providers). First, small practices continue to provide primary care for a significant proportion of the population. More than half of primary care office visits occur in small practice settings [3–5]. Second, small practices face daunting challenges in redesigning their system and care processes to meet regulatory requirements for practice transformation. Most small practices lack the resources and staff expertise related to information systems, goal setting, data analysis, and practice redesign to coordinate a complex set of multilevel system changes without external assistance [6]. One implementation strategy that may effectively overcome these barriers is practice facilitation (PF) [7, 8].

PF provides external expertise to help organizations make meaningful practice changes that are tailored to local context and in accord with current practice transformation models like the chronic care model (CCM) and patient-centered medical home (PCMH) [9, 10]. Implementation science literature suggests that PF’s emphasis on building organization capacity to adapt clinical evidence to the specific circumstances of the practice environment may be more effective in achieving sustainable improvements in guideline-concordant care and patient outcomes in primary care settings compared to single component (e.g., audit and feedback alone) and other multicomponent guideline implementation strategies (e.g., learning collaborative) [7, 8, 11–22]. A recent systematic review of PF interventions demonstrated that primary care practices with the support of a facilitator are almost three times more likely to implement evidence-based guidelines compared with usual care practices [7]. Despite this evidence, the effectiveness of PF remains largely untested in small primary care practices, especially those that serve vulnerable and underserved minority populations.

HealthyHearts NYC (HHNYC), one of seven cooperatives funded through the EvidenceNOW initiative, is a stepped-wedge cluster randomized controlled trial designed to evaluate the impact of PF compared to usual practice on implementation of the Million Hearts ABCS treatment guidelines in 250 small primary care practices, where a significant proportion of vulnerable populations in NYC receive care.

The specific aims of the HealthyHearts NYC project are to (1) compare the effect of PF versus usual care on ABCS outcome measures; (2) identify baseline organizational characteristics (e.g., site level adaptive reserve, organizational change capacity) that are associated with intervention outcomes; (3) use qualitative methods to assess barriers and facilitators to implementing practice change and achieving ABCS outcomes among high- and low-performing practices in the study; and (4) evaluate the intervention-specific costs per patient and per site.

## Methods

### Study setting

HHNYC is a partnership between New York University School of Medicine (NYUSOM) and the NYC Department of Health and Mental Hygiene (NYCDOHMH) Primary Care Information Project (PCIP), which has over 1300 small- to medium-sized primary care practices in its regional extension center [23]. The study is being conducted in small practices in NYC that are part of PCIP’s practice network. NYC has the largest (8.5 million) and most diverse population of any city in the US (26 % Hispanic, 26 % black, and 13 % Asian) [24]. Although NYC has achieved substantial decline in CVD-related deaths, heart disease still remains the number one cause of death and significant disparities by income and race/ethnicity persist. Residents of NYC’s poorest neighborhoods, where many of the study small practice sites are located, consistently have higher mortality rates from almost all diseases, including CVD, compared with residents in its wealthiest neighborhoods [25, 26]. For example, hypertension-related death rates among black New Yorkers are four times higher than among white New Yorkers (35 vs. 9 per 100,000 adults). Additionally, patients in PCIP network practices with a greater proportion of low income and minority patients in their panels are less likely to achieve target measures for blood pressure control and smoking cessation measures [27].

### Study design

HHNYC is a stepped-wedge cluster randomized controlled trial that is designed to evaluate the implementation process and the impact of PF (intervention) compared to usual practice (control), on implementation of the Million Hearts ABCS treatment guidelines in 250 primary care practices. All practice sites begin as part of the control condition and are block-randomized into four waves with each wave beginning 3 months after the start of the prior wave and lasting for 12 months (see Table 1). All clusters eventually receive the intervention, and outcomes will be measured every 3 months in all clusters. We anticipate, conservatively, a 15 % attrition rate; therefore, we have enrolled 290 sites to ensure that enrollment of the required 250 sites is achieved at the end of the study. This study has received Institutional Review Board approval by the New York University School of Medicine.

**Table 1:** Stepped-wedge study design

	Year 1 (months)				Year 2 (months)				Year 3 (months)			
Clusters	3	6	9	12	15	18	21	24	27	30	33	36
	Time period											
1 (80 sites)		C	X	X	X	X	F	F				
2 (70 sites)		C	C	X	X	X	X	F	F			
3 (70 sites)		C	C	C	X	X	X	X	F	F		
4 (70 sites)		C	C	C	C	X	X	X	X	F	F	

*C* control period, *X* intervention period, *F* follow up period

### Study site eligibility

We recruited practices from PCIP’s practice network, which was established in 2005 to improve health outcomes in low-income communities in NYC. This network includes over 1300 small- to medium-sized practices with significant numbers of Medicaid or uninsured patients. To be eligible, practice sites were required to have <10 full-time equivalent (FTE) healthcare providers (physician, nurse practitioner, physician assistant); have implemented an electronic health record (EHR) for at least 1 year; sign an agreement with PCIP to participate in PCIP’s Hub Population Health System, an EHR query architecture; have no immediate future plans to participate in a CVD-related quality improvement initiative; have no plans to change EHRs in the next 18 months; be willing to identify a site coordinator to work with study staff on all aspects of the intervention; and be located in NYC [28]. We will recruit 40 of the sites that are enrolled in the study to participate in the qualitative assessment described below. For the qualitative interviews, sites will be chosen to ensure representation across different geographic locations in NYC and practice size.

### Patient eligibility

Data is being abstracted for patients who: (a) have at least one of the ABCS risk factors (i.e., eligible for aspirin, has a diagnosis of hypertension, meets criteria for blood cholesterol management, and/or is a current smoker); (b) have received care at the site in the last 12 months; and (c) are age >18 years. Patients eligible for aspirin are those with a documented ICD (ICD-9/10) code for ischemic vascular disease in the last 12 months. Similarly, patients with a diagnosis of hypertension must have a documented ICD code for the targeted risk factor. Patients with hyperlipidemia based on an ICD code or who otherwise meet criteria for cholesterol management are identified by laboratory results for LDL cholesterol as well as ICD codes. Smokers are identified by documentation of current smoking status in the EHR (e.g., meaningful use measure) during the past 24 months.

### Site recruitment

PCIP focused recruitment on small practices in their network, which comprises over 1300 independent primary care sites in NYC. After a preliminary assessment of eligibility, the sample was reduced to a pool of 547 practices that are focused solely or primarily on adult primary care and currently using an EHR with data sharing capacity. Recruitment started with targeted invitation letters to an initial group of practices via post and email, which included a link to a recorded informational webinar. Simultaneously, PCIP conducted telephone outreach to determine practice site interest, to conduct a more detailed eligibility screening survey, and to secure a verbal commitment to participate. Once practices agreed to participate, PCIP and NYU co-hosted multiple in-person “kickoff” events to educate sites about program details. At these events, eligible practices were asked to sign the study participation agreement. In order to eliminate the risk of contamination between multiple sites in the same practice, only one site per target practice was permitted to join the study.

### Conceptual framework

**Fig. 1:**
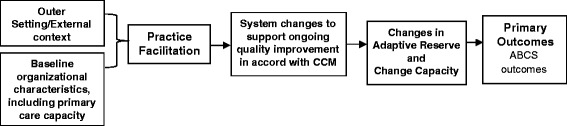
Conceptual framework

The study draws on Damshroeder’s Consolidated Framework for Implementation Research (CFIR) and Solberg’s framework for improving medical practice [29, 30]. Our model posits that improvements in provider adherence to guideline-recommended care and ABCS-related clinical outcomes are a function of baseline external context (e.g., external policies and incentives), the baseline organizational/site characteristics, including change process capability and adaptive reserve, the intervention strategies (i.e., practice facilitation), and the multilevel changes in care processes and systems (e.g., EHR enhancements) that are employed as a result of the intervention strategies (Fig. 1). The model postulates that PF, and the resulting system changes, will enhance ABCS outcomes through changes they produce in adaptive reserve and change process capability [31, 32].

### Intervention components

#### Practice facilitation protocol

The duration of the intervention is 12 months for each wave, and it is being implemented at the site level. PF comprises the following components: (1) onsite visits by the practice facilitator with the medical director and/or staff twice in the first month and then monthly thereafter; (2) expert consultation (i.e., quarterly webinars and “ask the expert” email consultant service); and (3) facilitated opportunities for shared learning across intervention sites (e.g., peer-to-peer collaborative calls) [8]. The practice facilitator’s main role is to coordinate and facilitate meetings with study sites and to assist primary care practices in setting practice change and performance goals and in developing strategies for implementing evidence-based components of the models for practice transformation, including CCM, PCMH, and Bodenheimer’s 10 building blocks of primary care [9, 10, 33]. For this purpose, a quality improvement (QI) manual informed by these models was developed by PCIP to drive delivery of the intervention. PFs also share best practices across sites, train practice staff on QI processes and methods to help them develop robust QI capacity (e.g., workflow mapping, generating performance reports), assist teams in testing system changes and interpreting outcomes, and assist with generating data for performance feedback and Plan-Do-Study-Act (PDSA) cycles. The practice facilitator conducts phone and email exchanges with the sites as needed between site visits during the 12-month intervention period.

#### Practice facilitator training and supervision

All practice facilitators received training at the State University of New York at Buffalo Practice Facilitator Certificate Program, a 92-h course with 13 weekly 1.5-h live online classes plus a 40-h fieldwork practicum and 26 h of reflective learning [34]. Twenty-one modules, based on AHRQ’s Practice Facilitation Handbook, cover the full range of topics for trainees to achieve the four core competencies for practice facilitation: data use to drive improvement; interpersonal skills; Health IT optimization; and QI and change management methods (e.g., Plan-Do-Study-Act) [35]. PCIP supplemented the Buffalo program with an additional 20 h of training that focused on increasing knowledge of the ABCS practice guidelines and optimizing the use of the EHR, including creating registries and dashboards, activating clinical decision support systems and other practice change components that correspond to the PF activities in the PCIP QI manual. ABCS toolkits were developed for the practice facilitators to follow that correspond directly to the training content. Three PF managers meet weekly with up to five facilitators each to provide oversight and opportunities for problem solving, identify additional training needs or site specific resources, and review the structured weekly activity reports and monthly narrative reports.

### Evaluation

Using a mixed methods approach, we will conduct a process and outcomes evaluation of five interrelated domains: (1) primary outcome (reaching clinical goals for ABCS measures); (2) secondary outcome (change in practice capacity); (3) internal context; (4) external context; and (5) implementation fidelity (PF activities, practice change). We will also assess the cost of the intervention.

#### Outcome measures and data source

The primary outcome is the proportion of patients who reach clinical goals for hypertension, blood cholesterol management, aspirin use, and smoking cessation assistance (Table 2). The measures were defined by AHRQ EvidenceNOW and finalized through a collaborative process across all the seven funded groups. We will collect data for the 12-month period prior to implementing the intervention, during the 12-month intervention period, and then 6 months post-intervention. These data will be extracted from the EHRs of all 250 participating sites using PCIP’s Hub, a query architecture that enables execution of queries in independent EHRs of the participating practices with data subsequently transmitted to a secure centralized clinical data repository [28]. The Hub provides a platform for HHNYC to extract numerator and denominator data for the ABCS measures. The underlying data quality/integrity of practices connected to the Hub is based on assessment of documentation patterns at the practices (e.g., completeness of use of routine structured data fields such as demographics, diagnosis, vital signs, and smoking status) as well as utilization of electronic laboratory interfaces, to ensure laboratory results data are available in electronic format [36].

**Table 2:** Outcome measures, data source, and data collection timeline

Domain	Measures	Data source	Administration
Primary outcomes	• Aspirin: percent of patients aged 18 years and older with ischemic vascular disease (IVD) with documented use of aspirin or other antithrombotic. • Blood pressure management: 1) Percent of patients aged 18–85 who had a diagnosis of hypertension (HTN) and whose blood pressure (BP) was adequately controlled (<140/90). 2) Percent of patients aged 60–85 without diabetes (DM) or chronic kidney disease (CKD) with controlled BP (<150/90), 3) Percent aged 18–85 with DM or CKD with controlled BP (<140/90) • Cholesterol management: 1) Percentage of patients aged >21 diagnosed with ASCVD who are on statin therapy. 2) Percentage of patients aged >21 with history of LDL >190 mg/dL without ASCVD who are on statin therapy. 3) Percentage of patients aged 40–75 with diabetes without ASCVD and LDL 70–189 mg/dL who are on statin therapy • Smoking cessation support: Percentage of patients aged 18 years or older who were screened about tobacco use one or more times within 24 months and who received cessation counseling intervention if identified as a tobacco user.	EHR	Baseline, quarterly during intervention period, at the end of intervention (12 months), and 6 months post intervention
Secondary outcome: practice change capacity	• Organizational change process capacity • Adaptive reserve	Provider and staff survey	Baseline, at the end of intervention (12 months) and 6 months post intervention

The secondary outcome is changes in primary care practice capacity captured through the use of the change process capacity questionnaire and the 23-item adaptive reserve scale [31, 32]. These measures will be obtained through provider and staff surveys at three time points: baseline, immediately following the 12-month intervention and 6 months post-intervention.

### Process evaluation measures and data collection methods

The process evaluation will have four components: internal practice context; external healthcare context; implementation fidelity; and cost (see Table 3).

**Table 3:** Process measures, data sources and timeline for data collection

Domain	Measures	Data sources	Administration
Internal practice-level context	• Staffing/FTEs • Payer mix • Patient demographics • Baseline integration practice transformation components (population management) • PCMH certification	• Practice survey • Site visits and key informant (KI) interviews with sample of providers and staff	• Surveys at baseline, 12 and 18 months • Site visits and KI interviews baseline and 12 months
External context	• Regulatory and financial environment (e.g., pay-for-performance, value based payment)	• Provider surveys • KI interviews with sample of practice site providers and staff	• Ongoing
Implementation fidelity	• PF activities: dose, intensity and mode of delivery of PF intervention Practice change: Extent to which practice change components are implemented and in use	• PF activities: Web-based PF tracking system, Webinar registration and evaluation and collaborative call attendance • Practice change: practice change survey and site visits/KI interviews	• Ongoing PF tracking using saleforce.com • Practice change survey monthly
Cost	• Implementation and intervention cost	• Cost data collection template embedded in PF tracking system	• Ongoing and reviewed monthly

#### Internal and external context

These domains will be assessed using both qualitative and quantitative data. The lead clinician or lead practice administrator will be asked to complete a survey (in addition to the survey described previously to assess primary care capacity) that will assess practice characteristics (e.g., use of EHR, FTEs, PCMH status, patient demographics). The survey will be conducted at baseline, 12 and 18 months. In order to obtain a deeper understanding of internal and external context, we will conduct semi-structured interviews with at least one provider and one staff person at 40 sites spread over different waves. We will visit half of these sites early in their implementation of the intervention and again toward the end of the intervention period. The interviews will focus on the practice-specific context, the local and state health policy environment, and experience with practice facilitation. We will visit the other 20 sites one time post-intervention. These sites will be chosen to represent variability in response to the intervention in terms of changes in patient outcomes.

To gain a broader perspective on external health care context and ongoing and emerging practice transformation programs in New York State and nationally, we will conduct semi-structured interviews with 10 key informants who represent leaders in this area from the city and state.

#### Implementation fidelity

We will assess two core measures of fidelity: (1) PF activities (i.e., to what extent is the PF protocol adhered to in terms of practice visits and provider participation in webinars and collaborative calls?) and (2) practice change (i.e., to what extent is the change package, defined primarily by CCM and PCMH, implemented as specified?).

1. Practice facilitation activities: The primary outcome measure for PF activities is the frequency, duration, and mode of contact. PF activities are measured with data from the web-based software tool (Salesforce.com) that PCIP is currently using to track their practice facilitators’ activities. Each site has a separate page in Salesforce that includes the relevant elements for documenting PF activities and practice change components. Practice facilitators complete documentation in Salesforce after every visit including the time spent at the practice site, who the practice facilitator met with at the sites, what activities were discussed, outcomes of the visits, and agreed upon next steps (e.g., set date for upcoming visit). We will also track provider participation in other components of the PF intervention (e.g., webinars).
2. Practice change: We will also use Salesforce to assess our targeted practice changes (e.g., use of clinical decision support). Practice facilitators complete a survey, which is embedded in Salesforce, after each visit (i.e., monthly). The survey uses a 6-point scale (not yet educated; educated/PDSA; educated/using; educated/not using/deferred; educated/not using/refused; and not applicable) to assess practice change components that are the target of the intervention and correspond to the main elements of PCIP’s QI manual, which we have mapped to CCM and PCMH [9, 10]. Facilitators will also complete monthly narrative reports that will provide an overview of all implementation activities, including perceptions about what components of the intervention did and did not work and approaches to adaptation of intervention components.

#### Cost

We will apply a practical, cost assessment methodology developed by Ritzwoller to assess the implementation costs of the intervention [37]. This approach provides data on what resources would be needed to implement and replicate the intervention. To determine the implementation and intervention costs, we will collect resource costs based on health professionals’ time requirements (including training), the facilitator’s time, IT costs, and needed supplies or equipment. We will aggregate the data across individual cost capture templates based on the tasks and timeline of the project. Intervention-specific data are defined by asking the question “would this resource be needed to deliver the intervention in practice?” After intervention specific resources are differentiated from research and development, total intervention costs per patient and per practice can be calculated. We will also conduct sensitivity analyses to estimate the range of intervention costs in a variety of settings and circumstances.

### Analysis plan

#### Aim 1

The primary outcomes are the proportion of patients who reach clinical goals based on national guidelines for (a) aspirin use, (b) HTN control, (c) blood cholesterol management, and (d) smoking cessation assistance at the end of the intervention (12 months) period and 6 months post-intervention. Each of the four hypotheses referring to the four primary outcomes will be tested separately with a two-sided test with *α* = 0.05, i.e., no adjustment for multiple testing will be applied. The analysis of effect of PF on the ABCS outcome measures in the context of a stepped-wedge design will be based on a generalized linear mixed model (GLMM). In particular, to assess the intervention effect, we will use a Poisson model with random site and provider within site effects. Each model will be as follows:

1 $$ \log \left({C}_{it}\right)=\mu +{\beta}_1t+{\beta}_2{I}_{it}^{PF}+{\beta}_3{I}_{it}^{PF}\left(t-{s}_i\right)+\gamma {Z}_i+{b}_i+ \log \left({E}_{it}\right), $$

where *C*_*it*_ is the number of patients in site *i* who (a) had received aspirin, (b) had controlled blood pressure, (c) had controlled cholesterol, or (d) received smoking cessation counseling during period *t*, for *t* ∈ (0, 1, …, 11). Each period is 3 months, and *t* = 0 is the baseline quarter. *I*_*it*_^PF^ is an indicator variable, and *I*_*it*_^PF^ = 1 if site *i* has been assigned to the PF intervention at period *t*, *I*_*it*_^PF^ = 0 otherwise. *s* is the time period when the PF intervention begins for site *i. Z* is a site-level indicator variable, *Z* = 1 if site *i* has three or more providers, *Z* = 0 otherwise. *E*_*it*_ is the number of patients at site *i* who were eligible to be counted during period *t*. The eligible pool of patients *E*_*it*_ will be different for each outcome and has been described in detail in Table 1. Log(*E*_*it*_), considered the “offset” in the Poisson regression model, is a random effect for site *i* with mean 0 and variance *σ*_*b*_^2^. The estimation via model (1) takes into account a general time trend and allows for the intervention effects to grow over time following implementation of the intervention.

Our primary outcome of interest for each of the four models is proportion of patients who meet clinical goals (described above) at 12 months. In each model, this effect (after taking into account general time trends) would be captured by *β*_2_ + 3*β*_3_. For the four primary outcomes, we will conduct tests with these null and alternative hypotheses: *H*_0_ : *β*_2_ + 3*β*_3_ = 0 vs. *H*_*A*_ : *β*_2_ + 3*β*_3_ ≠ 0. The models will be fit using the LME4 package using R software.

### Power/effect size analysis

With the pre-specified sample size of 250 sites and design (wedge design with four strata/waves starting 3 months apart), we investigated the intervention effects under each of the primary hypotheses in Aim 1, that are detectable with at least 80 % power of a two-sided significance test with *α* = 0.05 (Appendix). Conversely, we assessed the power to detect effects that are clinically meaningful and realistic. For each of the four outcomes, we are interested in the proportion of eligible patients in a site who meet guideline recommendations for ABCS clinical care in the fourth quarter of the PF intervention (i.e., month 12 of the intervention) relative to compliance proportions at baseline.

Using conservative (i.e., low) eligibility levels of an average of 50 patients per physician per quarter, we will have over 80 % power to detect a 2.0 % change, assuming a starting compliance rate of 20 % (increasing from 20.0 to 22.0 %). Finally, we have little information to estimate the current levels of variation between sites (*σ*_*b*_^2^). However, the simulations suggest that power does not decrease when the assumed variance is increased from 0.10 to 0.25 on the log scale. If in fact the variance is lower, closer to 0.01 or 0.05, power is increased substantially to over 95 %.

#### Aim 2

We will identify baseline organizational characteristics (e.g., site level adaptive reserve, organizational change capacity) that are associated with primary outcomes. This will be accomplished by including separately each organizational characteristic in the main models used under Aim 1 as a main effect and in interactions with the PF indicator. We will conduct a further analysis to explore the possibility that changes in organizational level factors mediate the effect of the intervention on the primary compliance outcomes. Mediation analysis methods in the context of a stepped-wedge design have not been well developed. We will conduct a preliminary analysis to assess if the intervention is associated with changes in site level adaptive reserve, organizational change capacity, and practice changes (assessed with the practice change survey). If there is a detectable effect of the intervention on the organizational level factors, we will assess whether the changes in these organizational level factors are associated with changes in the primary compliance outcomes using the Baron and Kenny “product method” as a starting point of the analysis [38]. If necessary, we will explore more complex structural equation models (SEM) adapted for Poisson outcomes as well as more recently developed causal inference mediation methods to evaluate the mediation.

#### Aim 3

We will use qualitative methods to conduct a more in-depth assessment of internal and external context including barriers and facilitators (among high and low performers) to implementing practice change and achieving ABCS outcomes. The transcriptions of the interviews and focus groups will be coded using Atlas.ti. The coding scheme will be developed by the evaluation team to focus on key dimensions identified both a priori (i.e., from the interview and focus group protocols) and those that emerge during site visits, interviews, and focus groups. Two coders will independently code at least 10 interviews and all focus groups. Double coding will continue until adequate inter-rater reliability is achieved.

Most of the quantitative data will be analyzed descriptively to provide documentation and description of the practices, the implementation components, fidelity, and context. Using the score calculated from the practice change survey, we will evaluate changes over time in implementation of the practice change components (i.e., one component of implementation fidelity) with repeated measures ANOVA. We will also explore the association between implementation fidelity and primary outcomes (Aim 1 and 2 analyses).

## Discussion

Half of the US adult population has one or more preventable risk factors for cardiovascular disease (CVD) including hypertension (HTN) and hyperlipidemia, but only 10 % are meeting all of their clinical goals [39]. As a result, CVD remains the number one cause of death in the US [40]. A recent analysis by Farley and colleagues concluded that optimal implementation of clinical guidelines for treating HTN and hyperlipidemia could prevent up to 100,000 deaths per year [41]. Yet, less than half of the patients with one or more CVD risk factors are receiving guideline recommended care [42–45].

HHNYC aims to fill this current research-to-practice gap by evaluating the effectiveness of practice facilitation as a quality improvement strategy for implementing evidence-based guidelines for reducing CVD-related risk factors in primary care practices in New York City. The specific focus is to improve adherence to the ABCS, with a long-term goal of creating a robust infrastructure to disseminate and implement patient-centered outcomes research (PCOR) findings in primary care practices and improve practices’ capacity to implement other PCOR findings in the future.

The study findings have the potential to accelerate the tempo of implementation and dissemination of evidence-based strategies for addressing CVD risk factors in primary care settings serving populations with higher risks of CVD-related mortality.

There are several potential methodological challenges that may be encountered during the project. First, obtaining high survey response rates from the providers can be challenging. The project team has extensive experience conducting provider surveys with the target study sites with good results. We will incorporate several strategies that have been effective in previous research in the current study sites including using financial incentives [46, 47]. Second, it is likely that we will observe variation in implementation fidelity. We have defined the core elements of the intervention; however, based on our previous experience and review of the current implementation literature, we acknowledge that adaptations to the unique practice context will be necessary. This is an inherent component of practice facilitation and is meant to enhance adoption and sustainability. We will use rigorous methods to measure implementation fidelity of core elements and will also document adaptations to enhance external validity. Finally, unforeseen changes in the external context of health care delivery may affect implementation outcomes (e.g., changes in Medicaid reimbursement). The practice context in NYC is similar to that nationally, with simultaneous implementation of numerous practice transformation programs and projects. Notable statewide activities include the Medicaid PCMH incentive program to expand primary care capacity and improve care coordination, especially for patients with chronic disease; support of two state-designated regional extension centers to promote adoption of electronic health records (EHRs); the Medicaid and Medicare meaningful use incentive programs; and, recently, the $6 billion Delivery System Reform Incentive Payment Program (DSRIP), which creates new regional collaborations to reduce avoidable hospitalizations among Medicaid patients [48, 49]. Programs and funding related to practice transformation will continue to evolve. Our plan to collect data on external context via key informant interviews with Steering Committee members and pre and post intervention provider surveys will allow us to analyze for any historical threats to internal validity.

## Abbreviations

ABCS, aspirin, blood pressure, cholesterol, smoking cessation; AHRQ, Agency for Healthcare Research and Quality; CCM, chronic care model; CFIR, Consolidated Framework for Implementation Research; CVD, cardiovascular disease; EBPG, evidence-based practice guidelines; EHR, electronic health records; FTE, full-time equivalent; GLMM, generalized linear mixed model; HHNYC, HealthyHearts New York City; NYC, New York City; NYCDOHMH, New York City Department of Health and Mental Hygiene; NYUSOM, New York University School of Medicine; PCIP, Primary Care Information Project; PCMH, patient-centered medical home; PCOR, patient-centered outcomes research; PDSA, Plan-Do-Study-Act; PF, practice facilitation; QI, quality improvement; SEM, structural equation models

## Appendix

### Power/effect size analysis

With the pre-specified number of sites (250) and design (wedge design with four strata starting 3 months apart, we investigate the intervention effects under each of the primary hypotheses in Aim 1, that are detectable with at least 80 % power of a two-sided significance test with *α* = 0.05. Conversely, we assess the power to detect effects that are clinically meaningful and realistic.

For each of the four outcomes, we are interested in comparing the proportion of eligible patients in a physician’s panel who meet compliance (i.e., appropriate blood pressure level, appropriate cholesterol level, indication that aspirin was recommended, and indication of referral for smoking cessation) in the third quarter after PF intervention with the compliance proportion at baseline. In particular, we are interested in the difference *d*_*π*_ = *π*_3_ − *π*_0_, where *π*_0_ is the baseline compliance rate, and *π*_3_ is the third period compliance rate. The proportions *π*_0_ and *π*_3_ will be estimated by *C*_0_/*E*_0_ and *C*_3_/*E*_3_, respectively, where *C* is the number of patients in compliance and *E* is the number of eligible patients. We will use the model to estimate the difference *d*_*π*_. However, because of the specific Poisson distributions of the data and the model that we will be using, the treatment effect is more naturally expressed as a ratio rather than a difference: *r*_*π*_ = *π*_3_/*π*_0_.

We conducted the simulations varying the ratio *r*_*π*_, but for the clarity of interpretation, we also present the effect as a difference *d*_*π*_. Our goal is to find the *r*_*π*_ ’ s (and *d*_*π*_ ’ s) that would allow 80 % power of a two-sided test for significance with *α* = 0.05. We simulate the outcomes based on different assumptions about baseline compliance rates, random effects (which are at the scale of log(*C*)), and patient eligibility numbers. For each set of assumptions, 2000 data sets were generated using the GLMM model given in Eq. (2).

2 $$ \log \left({C}_{jt}\Big|{d}_j\right)=\mu +{\beta}_1t+{\beta}_2{I}_{jt}^{\mathrm{PF}}+{\beta}_3{I}_{jt}^{\mathrm{PF}}\left(t-{s}_j\right)+\gamma {Z}_j+{d}_j+ \log \left({E}_{ijt}\right), $$

The rate ratio *r*_*π*_ is a function of baseline treatment effect (*β*_2_) and time-related treatment effect (*β*_3_) : *r*_*π*_ = exp(*β*_2_ + 2*β*_3_). For each of the 1000 data sets, we estimated the same model and tested the hypothesis that *β*_2_ + 2*β*_3_ (the effect size in the third quarter) was significantly different from 0. The proportion of significant results represented the power of the study under the specific set of assumptions.

For the purposes of simulation, we assumed 63 sites in each stratum, for a total of 252 sites. Preliminary simulations indicated that larger general time trends (*β*_1_) and larger number of patients in a physician panel both increased power. Based on this, our final simulations included very conservative assumptions of zero growth trend (*β*_1_ = 0) and average eligibility size (*E*_*jt*_) of 50 patients per physician at the practice. In the simulation, a general level of *E*_*j*_ for site *j* was generated from a Poisson distribution of mean 50 × the number of physicians at the site. The value of *E*_*jt*_ for a particular time period was calculated (and rounded to the nearest integer) as *E*_*ij*_ + *e*_*ijt*_, where *e*_*ijt*_ ~ *N*(mean = 0, sd = 2). For each site *j* during period *t*, log(*C*_*jt*_) was generated using equation (2). A value *C*_*jt*_, the number of patients in compliance for site *j* during period *t* was generated from a Poisson distribution with mean *C*_*jt*_.

Table 4 shows results after varying effect size parameters (*β*_2_ & *β*_3_), baseline compliance rate (*π*_0_), site-level random effect variance (*σ*_*d*_^2^), and patient eligibility levels (*E*). The current baseline compliance rates for the four measures are 49 % (blood pressure control), 33 % (LDL control for cholesterol), 35 %, (aspirin recommendation), and 20 % (smoking cessation referral). The number of patients that will be eligible in each 3-month period is based on an average physician roster of approximately 1,000 patients. Currently, 30 % of those patients have hypertension, 30 % have hyperlipidemia, and 20 % smoke. If we make a conservative estimate that 25 % of the patients will have an office visit per 3-month period, each eligible category will range from 50 to 75 patients. In Table 4, we use the lowest (most conservative) assumption of 50 patients, which reflects the likely size of the smoking group. At this level of eligible patients, we will have over 80 % power to detect a 2.2 % change, assuming a starting compliance rate of 20 % (increasing from 20.0 to 22.2 %) and between-site variance of 0.10 on the log scale.

As the number of eligible patients *E* increases, power to detect the same size change increases; for example, with the same effect (a 2.2 % change) there is 92 % power when 70 patients per period have an office visit. This means that we will have increased power for the hypertension, cholesterol, and aspirin measures compared to smoking cessation. In addition, power increases as the baseline compliance rate increases; for example, there is 91 % power to detect a 2.7 % change when the baseline compliance rate is 25 %. Finally, we have little information to estimate the current levels of variation between sites (*σ*_*d*_^2^). However, the simulations suggest that power does not decrease when the assumed variance is increased from 0.10 to 0.25 on the log scale. If in fact the variance is lower, closer to 0.05, power is increased substantially.
